# Factors Associated With Adverse Outcomes Following Duodenal Atresia Surgery in Neonates: A Retrospective Study

**DOI:** 10.7759/cureus.22349

**Published:** 2022-02-17

**Authors:** Koichi Deguchi, Yuko Tazuke, Rei Matsuura, Motonari Nomura, Hiroaki Yamanaka, Hideki Soh, Akihiro Yoneda

**Affiliations:** 1 Department of Pediatric Surgery, Graduate School of Medicine, Osaka University, Suita, JPN; 2 Department of Pediatric Surgery, Osaka Women's and Children's Hospital, Izumi, JPN; 3 Department of Pediatric Surgery, Aizenbashi Hospital, Osaka, JPN; 4 Department of Pediatric Surgery, Yao Tokushukai General Hospital, Yao, JPN; 5 Department of Pediatric Surgery, Kawasaki Medical School, Kurashiki, JPN; 6 Department of Pediatric Surgery, National Center for Child Health and Development, Tokyo, JPN

**Keywords:** pre-term, prematurity, low birth weight, duodenal atresia, complications

## Abstract

Objectives

There is limited evidence on the infants' postoperative complications who have undergone surgical repair of duodenal atresia and stenosis. This study aimed to identify the factors associated with poor surgical outcomes after the initial repair.

Methods

We retrospectively reviewed the data of 82 patients who underwent surgery for duodenal atresia and stenosis between January 1994 and December 2013 at our institution. Gestational age, birth weight, fetal growth, and other associated anomalies were recorded. Multivariate regression analyses were used to identify the factors associated with surgical outcomes, including postoperative complications and time to full oral intake.

Results

The median gestational age was 37.6 weeks, with 30 (37%) preterm (<37 weeks) and 11 (13%) early preterm (<34 weeks) infants. The median birth weight was 2531 g, with 27 (33%) patients < 2000 g and 10 (12%) patients < 1500 g. Postoperative surgical complications were identified in 18 (22%) cases, of which 12 (15%) required additional operations. Multivariate regression analysis revealed that a combination of very low birth weight (<1500 g) and early preterm was significantly associated with both surgical and non-surgical postoperative complications (p = 0.0028 and 0.021, respectively) and a prolonged time to full oral intake postoperatively (p = 0.013).

Conclusion

Very low birth weight and early preterm were significantly associated with postoperative complications and a prolonged time to full oral intake.

## Introduction

Congenital duodenal atresia and stenosis are common causes of congenital intestinal obstruction. Surgical treatment for duodenal atresia and stenosis is usually performed during the neonatal period. The survival rate of patients who undergo surgical treatment for duodenal atresia and stenosis has remarkably improved from 45% to 96% over the last 50 years due to improvements in diagnosis, neonatal intensive care, and surgical management [1].

Previous studies on patients who had undergone surgical treatment for esophageal atresia and congenital heart disease have reported that postoperative complications and mortality are associated more with a low birth weight than with normal birth weight [2-6]. Other studies also confirmed that preterm birth was one of the independent risk factors for severe postoperative complications in newborns who underwent surgery [7,8]. However, no studies have examined the incidence of postoperative complications and their underlying risk factors in patients who have undergone surgical repair of duodenal atresia and stenosis, except for the report by Escobar et al. [9]. Therefore, this study aimed to retrospectively analyze a cohort of patients who underwent surgical repair of duodenal atresia and stenosis to determine whether preterm and low birth weight patients were more vulnerable to poor postoperative outcomes.

## Materials and methods

Patient selection and data collection

A total of 89 patients underwent surgical treatment for duodenal atresia and stenosis between 1994 and 2013 at Osaka Women’s and Children’s Hospital, Izumi, Japan. After excluding seven patients who underwent laparoscopic surgery, the medical records of the remaining 82 patients were reviewed to retrospectively collect the following clinical data: estimated gestational age (GA), birth weight, antenatal diagnosis, sex, age at the initial operation, clinical presentation (including the type of duodenal atresia and stenosis and any other associated anomalies or chromosomal abnormalities), surgical approach, postoperative complications, and death within 90 days.

Primary and secondary outcome measures

The primary outcome measures were complications and deaths within 30 and 90 postoperative days, respectively. All postoperative complications were stratified as being either surgical or non-surgical and were graded I, II, IIIa, IIIb, IVa, IVb, or V according to the Clavien-Dindo classification system [10]. Respiratory complications and the need for prolonged respiratory support were excluded from the analysis to avoid the potential influence of an immature respiratory function.

The secondary outcome measures for poor surgical outcomes included delays in full oral intake and the length of hospital stay postoperatively. The length of hospital stay was adjusted by subtracting the number of days from a GA of 37 weeks and zero days to eliminate the potential influence of a lower GA.

Variables that were analyzed as potential risk factors for the above outcomes included sex, estimated GA, birth weight (BW), chromosomal abnormalities, other associated anomalies, fetal growth (small for gestational age [SGA], appropriate for gestational age [AGA], and large for gestational age [LGA]), operative time, bleeding, and postoperative morbidities.

Definitions

Very low birth weight was defined as birth weight < 1500 g. The term, preterm, and early preterm were defined as GA ≥ 37 weeks, 34-37 weeks, and <34 weeks, respectively. Fetal growth was categorized into three groups: SGA, AGA, and LGA. SGA and LGA were defined according to the Japanese neonatal anthropometric charts for GA at birth as birth weight below the 10th percentile and that above the 90th percentile, respectively [11].

Statistical analyses

Continuous variables were expressed as medians with ranges. Logistic regression analysis was used to assess the association of each factor with postoperative complications, which were expressed as the odds ratio (OR) with a 95% confidence interval (CI). Multicollinearity was assessed by multivariate logistic regression. Due to the significant multicollinearity in the statistical analyses and the significant overlap between very low birth weight and early preterm patients (10 of 11 patients, 91%), we combined these two variables for further statistical analyses. Time to full oral intake and the length of postoperative hospital stay were analyzed using the Kaplan-Meier method and the log-rank test. A Cox proportional-hazards model was used to identify the association of each factor with secondary outcomes, which were expressed as the hazard ratio (HR) with a 95% CI. All analyses were performed using the JMP Pro software, version 15.0 (SAS Institute, Cary, North Carolina, USA). Values of P < 0.05 were considered statistically significant.

This study was presented at the Pacific Association of Pediatric Surgeons (PAPS) 2015 Annual Meeting and reviewed by our institutional review board at the time, who waived the need for approval due to the retrospective nature of the investigation.

## Results

The preoperative features of the patients are shown in Table 1. The median GA was 37.6 weeks (range: 27.1-41.6), with 11 (13%) patients being early preterm (GA < 34 weeks). The median birth weight was 2531 g (range: 810-3775), with 10 (12%) patients having very low birth weight (<1500 g). Antenatal diagnoses of duodenal atresia and stenosis were established in 51 (62%) patients, and SGA was noted in 51 (62%) patients. Other associated congenital anomalies were present in 55 (67%) patients, and chromosomal abnormalities - the most common being Down syndrome - were present in 18 (22%) patients.

**Table 1:** The preoperative features of the patients with duodenal atresia and stenosis ^a^: Data are shown as median (range). GA: Gestational age; SGA: small for gestational age; AGA: appropriate for gestational age; LGA: large for gestational age.

Clinical features	N = 82
GA (weeks)^a^	37.6 (27.1-41.6)
Preterm, n (%)	30 (37%)
Early preterm < 34 weeks, n (%)	11 (13%)
Birth weight (g)^a^	2531 (810-3775)
Birth weight < 2000 g, n (%)	27 (33%)
Birth weight < 1500 g, n (%)	10 (12%)
Antenatal diagnosis, n (%)	51 (62%)
Sex (M:F), n	42:40
SGA:AGA:LGA, n	51:29:2
Associated anomalies, n (%)	55 (67%)
Chromosomal abnormalities, n (%)	18 (22%)
Cardiac anomalies, n (%)	25 (30%)

The details of the associated congenital anomalies are presented in Table 2. The associations between birth weight, GA, and fetal growth are summarized in Table 3. Overall, we confirmed a correlation between GA and birth weight: for example, most patients with a birth weight > 2000 g were term, whereas most patients with a birth weight < 2000 g were preterm.

**Table 2:** Details of associated anomalies Associated anomalies confirmed after birth and maternal events were shown in this table. VSD: Ventricular septal defect; PDA: patent ductus arteriosus; ASD: atrial septal defect; DORV: double outlet of the right ventricle; PLSVC: persistent left superior vena cava; RDS: respiratory distress syndrome; CDH: congenital diaphragmatic hernia.

Categories	Congenital anomalies	n	Details
Chromosomal		18	
	21 trisomy	16	
	18 trisomy	1	
	Ring chromosome 13	1	
Syndromes		3	
	VACTER association	1	
	Feingold syndrome	1	
	Rubinstein-Taybi syndrome	1	
Neurologic		3	
	Others	3	Microcephaly, cerebellar hypoplasia, hydrocephaly
Cardiovascular		49	
	VSD	9	
	Atrioventricular septal defect	8	
	PDA	7	
	Single ventricle defects	6	
	ASD	5	
	DORV	4	
	Asplenia/polysplenia/heterotaxy	3	
	PLSVC	3	
	Others	4	Transposition of the great arteries, tetralogy of Fallot, right aortic arch, coronary arteriovenous fistula
Respiratory		19	
	Pulmonary hypertension	7	
	Tracheomalacia	5	
	RDS	2	
	Tracheal stenosis	2	
	Others	3	Tracheal agenesis, lung agenesis, CDH
Gastrointestinal		55	
	Intestinal rotational abnormalities	16	
	Esophageal atresia	8	
	Annular pancreas	7	
	Anorectal malformations	7	
	Pancreatobiliary malformations	3	
	Meckel's diverticulum	3	
	Meconium plug syndrome	2	
	Gastrointestinal allergy	2	
	Gallbladder hypogenesis/agenesis	2	
	Others	5	Hiatal hernia, jejunal atresia, Hirschsprung's disease, superior mesenteric artery agenesis, portal vein agenesis
Genitourinary		9	
	Renal agenesis/hypogenesis	4	
	Undescended testis	2	
	Others	3	Hypospadias, multicystic kidney, neurogenic bladder
Skeletal		5	
	Sacrococcygeal anomalies	2	
	Others	3	Scoliosis, radioulnar synostosis, polydactyly
Maternal		8	
	Graves' disease	3	
	Umbilical cord ulcer	2	
	Others	3	ABO incompatibility, hypercoiled cord, premature rupture of membranes
Others		2	
		2	Transient abnormal myelopoiesis, hypothyroidism

**Table 3:** Associations between birth weight, estimated gestational age, and fetal growth ^a^: % of each subcategory. BW: Body weight; SGA: small for gestational age.

N = 82		Cases	%
Birth weight	BW > 2000 g	55	67
	BW < 2000 g	27	33
	BW < 1500g	10	12
Estimated gestational age	Term	52	63
	Preterm	30	37
	Early preterm < 34 weeks	11	13
Fetal growth retardation	nonSGA	31	38
	SGA	51	62
Chromosomal abnormalities	No	61	74
	Yes	21	26
Associated anomalies	No	27	33
	Yes	55	67
By subcategories		Cases	%^a^
BW > 2000 g		55	100
	BW > 2000 g, Term	45	82
	BW > 2000 g, nonSGA	26	47
	BW > 2000 g, Chromosomal abnormalities	11	20
	BW > 2000 g, Associated anomalies	34	62
BW < 2000 g		27	100
	BW < 2000 g, Preterm	20	74
	BW < 2000 g, SGA	22	81
	BW < 2000 g, Chromosomal abnormalities	10	37
	BW < 2000 g, Associated anomalies	21	78
	BW < 2000 g, Term, SGA, associated anomalies	7	26

The surgical outcomes are shown in Table 4. The median time to full oral intake was 22 days (range: 9-2719 days), and the adjusted length of postoperative hospital stay was 39.5 days (range: 7-1850 days). Intraoperative complications were documented in eight (10%) patients, which included intestinal injuries (four cases), misidentification of the proximal duodenum (one case), massive bleeding (one case), pancreatic duct injury (one case), and liver injury (one case). Postoperative surgical complications of grade II or higher occurred in 18 (22%) cases, of which 12 (15%) required reoperations (Table 4). Non-surgical complications of grade II or higher occurred in 20 (24%) patients. 

**Table 4:** The surgical outcomes of the patients with duodenal atresia and stenosis ^a^: Data are shown as median (range). ^b^: The adjustment is performed by subtracting the number of days from the gestational age of 37 weeks and 0 days from each period. DA: Duodenal atresia.

Surgical outcomes	N = 82
Ages at initial operation (days)^a^	4.0 (0-181)
Types of DA, n (%)	
Type 1	31 (38%)
Type 2	2 (2.4%)
Type 3	48 (59%)
Annular pancreas	9 (11%)
Intraoperative complications, n (%)	8 (10%)
Bleeding (mL)^a^	14.0 (0-122)
Operating time (min)^a^	148.0 (78-268)
Surgical complications, n (%)	18 (22%)
Reoperation	12 (15%)
Adhesive bowel obstruction	3 (3.7%)
Non-surgical complications, n (%)	20 (24%)
Time to full oral feeding (days)^a^	22.0 (9-2719)
Adjusted length of postoperative hospital stay (days)^a, b^	39.5 (7-1850)
90-day mortality, n (%)	3 (3.4%)

The details of surgical and non-surgical complications that occurred within 30 postoperative days are described in Table 5. Postoperative complications that required surgical intervention (grade IIIb) included adhesive bowel obstruction, anastomotic stricture, anastomotic leakage, surgical site infection, intestinal necrosis, and a missed second stricture. Delayed postoperative complications (after the initial 30 postoperative days) that required intervention were reported in eight patients, which included adhesive small bowel obstruction (five cases, of which four cases required reoperation), anastomotic stricture (one case), and pancreatobiliary malformation (two cases). Non-surgical complications of grade V caused all three deaths within 90 postoperative days (3.4%), which were due to respiratory infection, pulmonary hypertension, and life-threatening arrhythmia. These three patients had trisomy 18, cardiac anomalies (a complex of single atrium, double outlet right ventricle, common atrioventricular canal, and common atrioventricular valve regurgitation), and hemorrhagic shock due to umbilical cord ulceration, respectively.

**Table 5:** Postoperative complications within 30 days and comparisons according to the gestational age and birth weight Data are n (%). Postoperative complications are graded according to the Clavien-Dindo classification (>Grade II). ^a^: Total number of patients with either non-surgical or surgical complications. *: Statistically significant incidence of postoperative complications. GA: Gestational age; BW: birth weight.

Clavien-Dindo grade	Postoperative complications	Total patients, N = 82 (%)	GA < 34 weeks or BW < 1500 g, n = 12 (%)	GA ≥ 34 weeks and BW ≥ 1500 g, n = 70 (%)	p-value
All complications^a^ (Grades > II)		33 (40)	10 (83)	23 (33)	0.0025*
Non-surgical complications		20 (24)	6 (50)	14 (20)	0.062
Grade II	Enterocolitis	7 (9)	1 (8)	6 (9)	
	Cholestasis, medical treatment	9 (11)	2 (17)	7 (10)	
	Sepsis, biliary tract	1 (1)	1 (8)	0 (0)	
	Sepsis, catheter-related	1 (1)	1 (8)	0 (0)	
	Pneumonia	2 (2)	1 (8)	1 (1)	
Grade V	Life-threatening respiratory infection	1 (1)	0 (0)	1 (1)	
	Life-threatening arrhythmia	1 (1)	0 (0)	1 (1)	
	Pulmonary hypertension	1 (1)	0 (0)	1 (1)	
Surgical complications		18 (22)	8 (67)	10 (14)	0.0004*
Grade II	Anastomotic stricture	7 (9)	2 (17)	5 (7)	
	Surgical site infection, medical treatment	3 (4)	2 (17)	1 (1)	
	Anastomotic leakage, medical treatment	2 (2)	1 (8)	1 (1)	
	Gallstones	2 (2)	0 (0)	2 (3)	
	Gastroesophageal reflux	1 (1)	1 (8)	0 (0)	
Grade IIIb	Anastomotic stricture, reoperation	2 (2)	0 (0)	2 (3)	
	Anastomotic leakage, reoperation	2 (2)	1 (8)	1 (1)	
	Surgical site infection, reoperation	1 (1)	0 (0)	1 (1)	
	Intestinal necrosis, reoperation	1 (1)	1 (8)	0 (0)	
	Missed the second stricture	1 (1)	0 (0)	1 (1)	

The results of the logistic regression analyses to determine factors associated with postoperative complications are presented in Table 6. A combination of very low birth weight and early preterm was significantly associated with postoperative surgical complications (OR = 7.7, p = 0.0028) and non-surgical complications (OR = 4.9, p = 0.021). Twelve patients were categorized in this group (GA < 34 weeks or BW < 1500 g), and details of complications are described in Table 5. Overall, 83% (10 patients) had either non-surgical or surgical complications (all complications, Grades > II), and 67% (eight patients) had surgical complications (Grades > II). In contrast, in the remaining patient group (GA ≥ 34 weeks and BW ≥ 1500 g, n = 70), all complications (Grades > II) occurred in 33% and surgical complications in 14%: Both incidence rates were significantly lower compared to the former patient group (p = 0.0025 and 0.0004, Table 5). Chromosomal abnormalities were also significantly associated with non-surgical complications (OR = 3.4, p = 0.040, Table 6). There were no significant associations between other variables and postoperative complications.

**Table 6:** Results of the multivariate logistic regression analysis to predict postoperative complications (Grade > II) *: Statistically significant independent predictors of postoperative complications. GA: Gestational age; SGA: small for gestational age.

	Univariate analysis	Multivariate analysis		
	p-value	Odds ratio	95% CI	p-value
Surgical complications, Grades > II				
Gender	0.91			0.84
Chromosomal abnormalities	0.71			0.64
Associated anomalies	0.60			0.40
Birth weight < 1500 g or GA < 34 weeks	0.0027*	7.7	2.0-29.2	0.0028*
SGA	0.51			0.43
Non-surgical complications, Grades > II				
Gender	0.90			0.58
Chromosomal abnormalities	0.027*	3.4	1.1-10.7	0.040*
Associated anomalies	0.16			0.33
Birth weight < 1500 g or GA < 34 weeks	0.033*	4.9	1.3-18.6	0.021*
SGA	0.41			0.55

The results of the multivariate regression analyses to determine factors associated with the secondary outcome measures indicated that the presence of other associated anomalies and a combination of very low birth weight and early preterm were significantly associated with a prolonged time to full oral intake (HR = 0.53 and 0.39; p = 0.027 and 0.013, respectively, Table 7).

**Table 7:** Results of the multivariate Cox regression analysis to predict time to full oral intake *: Statistically significant independent predictors of delayed oral intake. GA: Gestational age; SGA: small for gestational age.

	Multivariate analysis		
	Hazard ratio	95% CI	p-value
Time to full oral intake			
Gender			0.50
Chromosomal abnormalities			0.47
Associated anomalies	0.53	0.30-0.93	0.027*
Birth weight < 1500 g or GA < 34 weeks	0.39	0.18-0.82	0.013*
SGA			0.63

**Figure 1:**
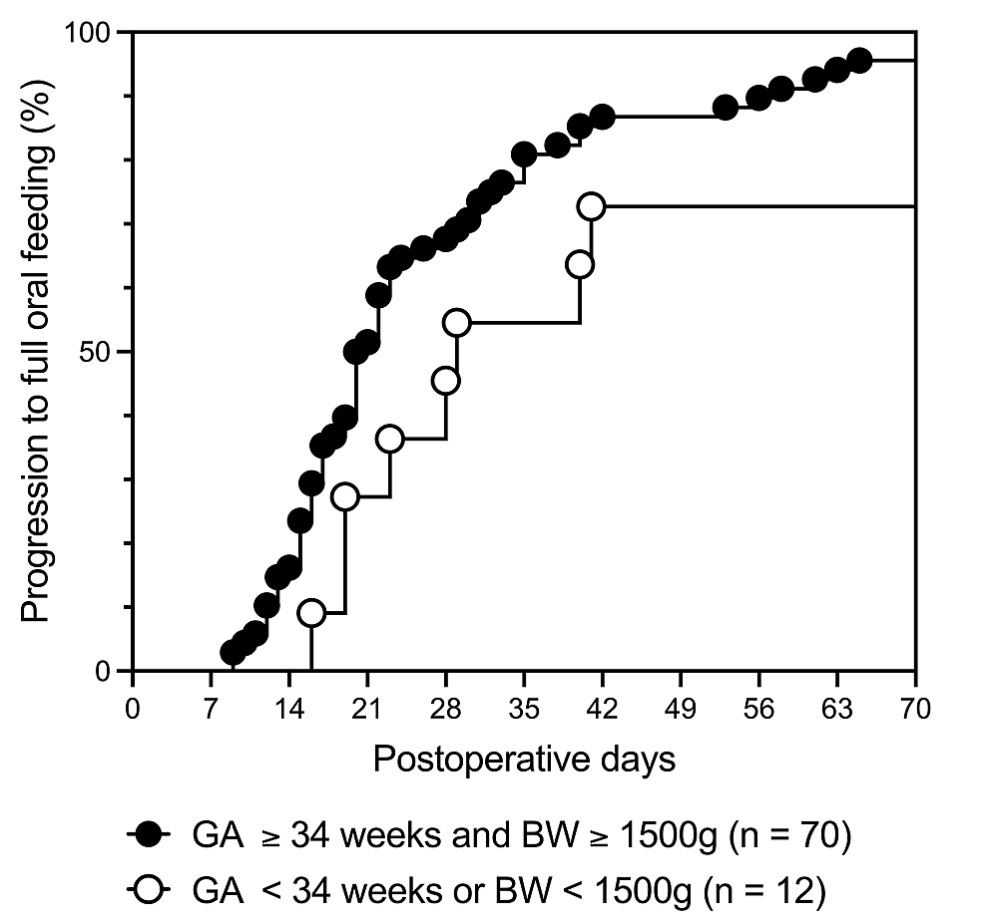
Kaplan-Meier curves illustrating the progression to full oral intake Kaplan-Meier curves illustrate the progression to full oral intake. The influence of low gestational age (GA) and birth weight (BW) suggested that if a patient was in GA ≥ 34 weeks and BW ≥ 1500 g (n = 70), then the median time to progression was 20.5 days (range: 9–120 days). However, if the GA < 34 weeks or BW < 1500 g (n = 12), then the median time to progression was 29 days (range: 16–2719 days) (p = 0.024, log-rank test).

Patients were further stratified based on GA and birth weight (Figure 1). Among patients with GA ≥ 34 weeks and birth weight ≥ 1500 g (n = 70), the median time to full oral intake was 20.5 days (range: 9-120), whereas in patients with GA < 34 weeks or birth weight < 1500 g (n = 12), the median time to full oral intake was 29 days (range: 16-2719, p = 0.024). There were no significant associations between any variables and a prolonged postoperative hospital stay (Table 8).

**Table 8:** Results of the multivariate Cox regression analysis to predict the adjusted postoperative hospital stay ^a^: The adjustment is performed by subtracting the number of days from a gestational age of 37 weeks and 0 days from each period. GA: Gestational age; SGA: small for gestational age.

Multivariate analysis	
Adjusted postoperative hospital stay^a^	p-value
Gender	0.68
Chromosomal abnormalities	0.22
Associated anomalies	0.36
Birth weight < 1500 g or GA < 34 weeks	0.76
SGA	0.45

Finally, we conducted two sub-group analyses to clarify the associations between a study period, trisomy 21, and surgical outcomes. First, we broke down our patient population into two epochs (the first half 10 years and the second half) and compared patient characteristics and outcomes (Table 9). The only statistically significant difference between the two groups was the rate of antenatal diagnosis, where the second 10 years group had a higher rate (41% vs. 77%, p = 0.0013). Surgical outcomes were not significantly different between the two groups. 

**Table 9:** Comparison of patient characteristics and outcomes divided by the first and second half study periods ^a^: Data are shown as median (range). ^b^: The adjustment is performed by subtracting the number of days from a gestational age of 37 weeks and 0 days from each period. *: A statistically significant characteristic between the two groups. GA: Gestational age.

	First half (1994-2003), n = 34	Second half (2004-2013), n = 48	p-value
GA (weeks)^a^	37.4 (30.9-41.3)	37.9 (27.1-41.6)	0.91
Preterm, n (%)	14 (41%)	16 (33%)	0.49
Early preterm < 34 weeks, n (%)	2 (6%)	9 (19%)	0.51
Birth weight (g)^a^	2575 (1110-3175)	2452 (910-3775)	0.26
Birth weight < 1500 g, n (%)	3 (9%)	7 (15%)	0.51
Antenatal diagnosis, n (%)	14 (41%)	37 (77%)	0.0013*
Sex (M:F), n	20:14	22:26	0.27
Associated anomalies, n (%)	26 (76%)	29 (60%)	0.16
Ages at initial operation (days)^a^	4.0 (1-181)	4.0 (0-101)	0.49
Operating time (min)^a^	140.0 (78-268)	148.0 (90-217)	0.58
Surgical complications, n(%)	8 (24%)	10 (21%)	0.79
Non-surgical complications, n(%)	6 (18%)	14 (29%)	0.30
Time to full oral feeding (days)^a^	22.5 (9-2719)	21.0 (10-99)	0.54
Adjusted length of postoperative hospital stay (days)^a, b^	39.0 (7-678)	43.5 (11-1850)	0.18
90-day mortality, n (%)	2 (6%)	1 (2%)	0.57

Second, we divided our patients according to the association with trisomy 21 and compared the patient characteristics and outcomes (Table 10). There were 17 patients associated with trisomy 21 and 65 patients without trisomy 21. We found no significant difference between the two groups regarding patient characteristics and outcome. As expected, patients with trisomy 21 required an extended hospital stay but were not statistically significant (74 days, p = 0.08). No mortality occurred in patients with trisomy 21.

**Table 10:** Comparison of patient characteristics and outcomes divided by the association with or without trisomy 21 ^a^: Data are shown as median (range). ^b^: The adjustment is performed by subtracting the number of days from a gestational age of 37 weeks and 0 days from each period. GA: Gestational age.

	With trisomy 21, n = 17	Without trisomy 21, n = 65	p-value
GA (weeks)^a^	37.1 (32.6-39.9)	37.6 (27.1-41.6)	0.39
Preterm, n (%)	8 (47%)	22 (34%)	0.40
Early preterm < 34 weeks, n (%)	3 (18%)	8 (12%)	0.69
Birth weight (g)^a^	2564 (1110-3100)	2514 (910-3775)	0.85
Birth weight < 1500 g, n(%)	2 (12%)	8 (12%)	0.99
Antenatal diagnosis, n (%)	9 (53%)	42 (65%)	0.41
Sex (M:F), n	5:12	37:28	0.06
Associated anomalies, n (%)	14 (82%)	41 (63%)	0.16
Ages at initial operation (days)^a^	4.0 (1-39)	3.5 (0-181)	0.50
Operating time (min)^a^	151 (78-217)	143 (88-268)	0.33
Surgical complications, n (%)	4 (24%)	14 (22%)	0.99
Non-surgical complications, n (%)	7 (41%)	26 (40%)	0.99
Time to full oral feeding (days)^a^	21.0 (12-99)	22.0 (9-2719)	0.34
Adjusted length of postoperative hospital stay (days)^a, b^	74.0 (7-1063)	39.0 (11-1850)	0.08
90-day mortality, n (%)	0 (0%)	3 (5%)	0.99

## Discussion

In this study, we retrospectively analyzed the data collected over 20 years from a single-institution cohort of 82 patients with congenital duodenal atresia and stenosis who were surgically managed during their neonatal period. Our results revealed that a combination of very low birth weight (<1500 g) and early preterm (<34 weeks) was significantly associated with postoperative surgical complications and non-surgical complications; this patient group experienced a high incidence of complications compared to the remaining patient group. The presence of chromosomal abnormalities was also significantly associated with postoperative non-surgical complications.

Prior studies have reported that survival after surgical repair of duodenal atresia and stenosis has improved by up to 96% over the last 50 years due to advances in neonatal surgical care [1,12]. In this study, the 90-day mortality rate over 20 years was 3.4%, which is comparable to previously reported rates in the literature [13,14]. The deaths that occurred in our cohort were attributed to trisomy 18, cardiac anomalies, and hemorrhagic shock due to umbilical cord ulceration. A previous study suggested a worse outcome in patients with trisomy 21; however, no such association was observed in our modern cohort (Table 10) [15].

Surgeries in premature neonates continue to be a challenge as these patients are at a greater risk of undesirable postoperative outcomes. Preterm or low birth weight neonates usually have immature organ function and are, therefore, highly vulnerable to invasive procedures and stressors [16]. However, studies on the incidence of postoperative complications and reoperation after initial repair of duodenal atresia and stenosis in premature neonates are lacking [9]. For precise classification, we have graded the postoperative complications of this cohort according to the Clavien-Dindo classification [10] and stratified them as being either surgical or non-surgical. We observed that very low birth weight and early preterm patients had significantly overlapping characteristics. A combination of these factors was significantly associated with postoperative surgical and non-surgical complications despite adjusting for the influence of other covariates. The overall incidence of postoperative complications in our study was 24% for non-surgical complications and 22% for surgical complications, which is similar to recent studies [14,17]. However, 83% had any complications (Grades > II), and 67% had surgical complications (Grades > II) in the patient group with very low birth weight and early preterm, which were significantly higher than the remaining counterpart (Table 5). The presence of chromosomal abnormalities was also significantly associated with postoperative non-surgical complications. On the contrary, sex, other associated anomalies, and SGA were not significantly associated with poor postoperative outcomes.

A retrospective study investigating intestinal atresia, including a duodenal atresia subset, showed that patients with a birth weight < 2000 g and those with associated anomalies were at an increased risk of prolonged hospital stay and mortality [18]. However, our results showed that patients with a birth weight < 1500 g or those born < 34 weeks of gestation were at an increased risk of postoperative morbidity rather than mortality.

Regarding secondary outcome measures, the presence of other associated anomalies and a combination of very low birth weight and early preterm were significantly associated with a prolonged time to full oral intake. No significant risk factors were identified for a prolonged hospital stay postoperatively. A previous study reported that delayed oral intake in patients with duodenal atresia and stenosis was significantly associated with GA < 35 weeks, congenital heart disease, and malrotation [14]; these results generally align with ours. However, the postoperative hospital stay adjusted for GA was not significantly associated with prematurity in our study. This indicates that the prolonged postoperative hospital stays in premature patients might have been influenced by their early GA rather than their vulnerability. This information may be useful during perinatal counseling.

A successful surgical approach for duodenal atresia and stenosis in a cohort that is particularly susceptible to postoperative morbidities has not yet been fully established [18]. Recently, some surgeons have advocated the “emergent” definitive surgical repair of duodenal atresia and stenosis even in premature neonates [9,19] based on recent reports that have suggested this procedure is associated with a low mortality rate in neonates. However, except in cases of concurrent malrotation with volvulus, congenital duodenal obstruction is not immediately life-threatening [20].

Our findings suggest that patients with severe prematurity are at an increased risk of postoperative complications and a prolonged time to full oral intake. Based on our results, we would like to endorse a slightly conservative approach for the management of stable patients with duodenal atresia and stenosis in which a thorough systematic search for any features that may affect their outcomes, along with proactive medical management to stabilize any physiological disturbances, is prioritized before taking a surgical decision. Very low birth weight and early preterm patients with duodenal atresia and stenosis may benefit from being transferred and treated in highly experienced tertiary neonatal centers comprising specialized surgeons and anesthesiologists. In cases where other associated anomalies that require earlier surgical correction - such as complex cardiac anomalies or esophageal atresia/tracheoesophageal fistulas - are present, we suggest that the duodenal obstruction be repaired later. During the initial anomaly intervention, these patients are maintained on parenteral nutrition without adverse liver effects [21].

The limitations of this study include its retrospective nature and its relatively small cohort size. On the other hand, the data from our study population were taken over a 20-year follow-up, in which consistent treatment guidelines (at a single center) were followed. There was no significant difference in patient characteristics and outcomes over the study period, except for the improved antenatal diagnosis in recent years (Table 9). As the information on premature patients who have undergone surgical management for congenital duodenal atresia and stenosis remains limited, we believe that our preliminary results, based on real-world clinical settings, are worth sharing among clinicians, patients, and their families. Well-designed studies with a longer follow-up period are required to establish the underlying risk factors for poor outcomes following surgical treatment for duodenal atresia and stenosis.

## Conclusions

In summary, we conducted a retrospective analysis of data from patients who underwent surgical treatment for duodenal atresia and stenosis to identify the predictors of poor postoperative outcomes. Based on our findings, we conclude that early preterm and very low birth weight are associated with a high incidence of postoperative complications and a prolonged time to full oral intake in patients with duodenal atresia and stenosis. Future studies should focus on the risk stratification among a cohort of infants with duodenal atresia and stenosis and optimization of the operative management strategies accordingly to mitigate the surgical risk of susceptible infants.
